# T Cell–Derived Granulocyte-Macrophage Colony-Stimulating Factor Contributes to Dry Eye Disease Pathogenesis by Promoting CD11b+ Myeloid Cell Maturation and Migration

**DOI:** 10.1167/iovs.16-20789

**Published:** 2017-02

**Authors:** Thomas H. Dohlman, Julia Ding, Reza Dana, Sunil K. Chauhan

**Affiliations:** Schepens Eye Research Institute, Massachusetts Eye & Ear Infirmary, Harvard Medical School, Boston, Massachusetts, United States

**Keywords:** cornea, dry eye, Th17 cell, granulocyte-macrophage colony-stimulating factor, monocyte/macrophage

## Abstract

**Purpose:**

Growing evidence suggests that granulocyte-macrophage colony-stimulating factor (GM-CSF) contributes to T helper 17 (Th17) cell–associated immunoinflammatory diseases. The purpose of this study was to evaluate the effect of T cell–derived GM-CSF on CD11b+ myeloid cell function in dry eye disease (DED).

**Methods:**

In a murine model of DED, quantitative real-time PCR and ELISA were used to measure GM-CSF expression at the ocular surface, and flow cytometry was used to enumerate GM-CSF producing Th17 cells. A granulocyte-macrophage colony-stimulating factor neutralizing antibody was used topically in vivo and in an in vitro culture system to evaluate the role of GM-CSF in recruiting and maturing CD11b+ cells. Clinical disease severity was evaluated after topical administration of GM-CSF neutralizing antibody.

**Results:**

In dry eye disease, GM-CSF is significantly upregulated at the ocular surface and the frequency of GM-CSF producing Th17 cells is significantly increased in the draining lymph nodes. In vitro neutralization of GM-CSF from CD4+ T cells derived from DED mice suppresses major histocompatibility complex II expression by CD11b+ cells and CD11b+ cell migration. Topical neutralization of GM-CSF in a murine model of DED suppresses CD11b+ maturation and migration, as well as Th17 cell induction, yielding a reduction in clinical signs of disease.

**Conclusions:**

T helper 17 cell–derived GM-CSF contributes to DED pathogenesis by promoting CD11b+ cell activation and migration to the ocular surface.

Dry eye disease (DED) is an extremely common condition that has a significant impact on patient quality of life.^1–3^ It is a multifactorial disease of the tears and ocular surface that results in visual disturbance, tear film instability, and damage to the ocular surface. Dry eye disease is now known to be mediated by the adaptive immune response.^4,5^ As part of the “afferent” arm of this response, antigen-presenting cells traffic from the ocular surface to draining lymph nodes,^6^ where they initiate the expansion and differentiation of effector T cells, particularly T-helper 17 (Th17) cells.^7,8^ These Th17 cells are then recruited to the ocular surface as part of the “efferent” or effector arm of the adaptive immune response, where they secrete proinflammatory cytokines that damage the ocular surface and further recruit and activate immune cells.^9^ However, many of the specific mechanisms comprising this Th17 cell effector response remain unknown.

T helper 17 cells play an important role in host defense against microbial pathogens, but have also been implicated in multiple immune-mediated inflammatory disorders^10^ and are now believed to be one of the primary effector immune cells in DED.^7,8,11^ They are characterized by their ability to produce IL-17A, IL-17F, IL-22, Chemokine (C-C motif) ligand (CCL) 20, TNF-α, and granulocyte-macrophage colony-stimulating factor (GM-CSF).^12^ Of these proinflammatory cytokines, GM-CSF is unique in that it is also a hematopoietic growth factor with multiple immune-modulating functions,^13^ making it a potential therapeutic target. In addition to its classic role in granulocyte and monocyte development, GM-CSF has been shown to increase the immunostimulatory activity of dendritic cells and stimulate dendritic cell production of proinflammatory cytokines including IL-6 and IL-23, which in turn promote Th17 cell development and effector function.^14–16^ There is evidence suggesting that Th17 cell–derived GM-CSF is required for the initiation of immune-mediated inflammatory diseases^17^; however, the precise role of GM-CSF in these conditions continues to be investigated.

In the present study, we investigated the role of GM-CSF in DED. Specifically, we hypothesized that in DED, Th17 cells produce GM-CSF and local neutralization of GM-CSF prevents the recruitment and maturation of CD11b+ antigen–presenting macrophages (derived from circulating monocytes), thus ameliorating disease.

## Materials and Methods

### Animals

We used 8- to 10-week-old female C57BL/6 mice from Charles River Laboratories, Inc. (Wilmington, MA, USA) in all experiments. Mice were housed and cared for in the Schepens Eye Research Institute animal vivarium following the guidelines of the ARVO Statement for the Use of Animals in Ophthalmic and Vision Research. All animal experiments and endpoints were overseen and approved by the institutional animal care and use committee.

### Dry Eye Model

As described previously,^18^ to induce DED mice were housed in a controlled environment chamber (CEC) for 12 days. The controlled environment chamber maintains constant air flow (15 L/minute) and keeps relative humidity below 20%. Naïve control mice were housed in room air conditions. Disease induction was confirmed and scored by corneal fluorescein staining (1% fluorescein; Sigma-Aldrich Corp., St. Louis, MO, USA) using the National Eye Institute (NEI) scoring scale.^18^

### Quantitative Real-Time PCR

Full-thickness cornea (two corneas per sample) and bulbar and palpebral conjunctiva (conjunctiva from two eyes per sample) were collected from naïve and DED mice and stored in reagent (TRIzol; Invitrogen, Carlsbad, CA, USA) at −80°C. After homogenization, RNA was isolated and reverse transcribed using an RNA purification kit (RNeasy Micro Kit; Qiagen, Valencia, CA, USA) and a cDNA synthesis kit (Superscript III; Invitrogen), respectively. Polymerase chain reaction was performed using a master mix (Taqman Universal PCR Master Mix; Applied Biosystems, Foster City, CA, USA) and the following primers: GM-CSF (Mm00438331_g1) and glyceraldehyde 3-phosphate dehydrogenase (GAPDH, Mm99999915_g1; Applied Biosystems). Comparative threshold (CT) values for GM-CSF and GAPDH were measured using a real-time PCR system (LightCycler 480 II System; Roche, Indianapolis, IN, USA). Comparative threshold GM-CSF values were normalized to GAPDH CT values (endogenous control), and the level of GM-CSF mRNA expression in DED mice relative to naïve mice was then calculated. Samples were run in triplicate.

### Enzyme-Linked Immunosorbent Assay

To measure secreted GM-CSF, full-thickness cornea (two corneas per sample) or bulbar and palpebral conjunctiva (conjunctiva from two eyes per sample) from naïve and DED mice were collected and stimulated with phorbol 12-myristate 13-acetate (PMA, 50 ng/mL; Sigma-Aldrich Corp.) and ionomycin (500 ng/mL; Sigma-Aldrich Corp.) in Roswell Park Memorial Institute (RPMI) medium (Thermo Fisher Scientific, Waltham, MA, USA) + 10% FBS for 24 hours at 37°C and 5% CO_2_. Supernatant was then collected and stored at −80°C with protease inhibitor (Sigma-Aldrich Corp.) until analyzed with a murine GM-CSF ELISA kit (MGM00, R&D Systems, Minneapolis, MN, USA). Samples were run in triplicate.

### Flow Cytometry

Submandibular and cervical draining lymph nodes were collected using jeweler's forceps (Katena Products, Inc., Denville, NJ, USA) and Vannas scissors (Storz; Bausch & Lomb, Rochester, NY, USA). Draining lymph nodes were homogenized to create single-cell suspensions, which were cultured with PMA (50 ng/mL; Sigma-Aldrich Corp.); ionomycin (500 ng/mL; Sigma-Aldrich Corp.); and protein transport inhibitor (0.7/100 μL media, GolgiStop; BD Biosciences, Franklin Lakes, NJ, USA) in RPMI (Thermo Fisher Scientific) +10% fetal bovine serum (FBS) for 5 hours at 37°C and 5% CO_2_. Cells were then stained with PE/Cy5-conjugated anti-CD4 (eBioscience, San Diego, CA, USA), fixed, and permeabilized using a commercially available fixation/permeabilization kit (eBioscience), then stained with FITC-conjugated anti-IL-17A (eBioscience) and PE-conjugated GM-CSF (BioLegend, San Diego, CA, USA). Using a flow cytometer (LSR II; BD Biosciences), 100,000 events were collected and analyzed using flow cytometry software (Summit v4.3; Dako Colorado, Inc., Fort Collins, CO, USA). For each experiment, *n* = 6 mice per group (1 mouse/flow sample). In vitro flow cytometry experiments are described below in “in vitro activation/proliferation assays” and in vivo neutralization flow cytometry experiments are described below in “in vivo GM-CSF neutralization and flow cytometry.”

### In Vitro Migration Assay

Draining lymph nodes were collected from 12-day-old DED mice and homogenized to create a single-cell suspension. We isolated CD4+ T cells using an anti-CD4 magnetic sorting kit (130-049-201, Miltenyi Biotec, Inc, San Diego, CA, USA) and 1 × 10^6^ CD4+ cells in 1 mL RPMI were stimulated overnight with plate-bound CD3 antibody. Over 90% of the sorted cells were CD4 T cells, with >95% viable cells. The next day, supernatant was collected and agitated at 4°C with either 2.5 μg/mL of anti–GM-CSF neutralizing antibody (505407; BioLegend) or rat immunoglobulin G (IgG 6-001-A; R&D Systems, Inc.) for 4 hours. During this incubation period, bone marrow from the femurs and tibias of naïve mice was collected, passed through a 70-μm cell strainer, and incubated with erythrocyte lysis buffer for 10 minutes. The resulting cell suspension was then stained with FITC-conjugated anti-CD11b (eBioscience, San Diego, CA, USA) and CD11b+ cells were isolated using a fluorescence activated cell sorter (FACS, MoFlo; Dako Cytomation, Carpinteria, CA, USA). Over 90% of the sorted cells were CD11b cells, with >95% viable cells. We added 400 μL of anti–GM-CSF or IgG-treated CD4+ supernatant to the bottom of a 24-well plate with 5-μm transwell pores. Then, 2 × 10^5^ CD11b+ cells in 100 μL media were added to the upper portion of the transwell and incubated at 37°C for 60 minutes, after which cells in the lower well were recovered and blindly enumerated using a hemocytometer. Each test condition (anti–GM-CSF and IgG treated) was run in triplicate.

### In Vitro Activation/Proliferation Assays

We isolated CD4+ T cells from DED mice as described above using an anti-CD4+ magnetic sorting kit (Miltenyi, Biotec, Inc.) and 5 × 10^5^ CD4+ cells in 500 μL RPMI were stimulated overnight with plate-bound CD3 antibody. The next day, supernatant was collected and agitated at 4°C with either 2.5 μg/mL of anti–GM-CSF neutralizing antibody (505407 BioLegend) or rat IgG (6-001-A, R&D Systems, Inc.) for 4 hours. We isolated CD11b+ cells from the bone marrow of naïve mice as described above and 3 × 10^5^ CD11b+ cells in 300 μL of anti–GM-CSF or IgG-treated CD4+ cell supernatant were cultured at 37°C for 24 hours. Cells were then collected and prepared according to one of two protocols for flow cytometry. To evaluate cell surface expression of major histocompatibility complex (MHC)-II, CD11b+ cells were collected and stained with PE/Cy5 conjugated MHCII (BioLegend). To evaluate cell proliferation by Ki67 expression, CD11b+ cells were fixed with 2% PFA and permeabilized with 0.7% Tween, then stained with AF647 conjugated anti-Ki67 (BioLegend). We collected 50,000 events per sample using a flow cytometer (LSR II; BD Biosciences) and analyzed using flow cytometry software (Summit v4.3; Dako Colorado, Inc.). Each analysis consisted of two groups (anti–GM-CSF and IgG), with three flow samples per group, with each flow sample derived from one well containing 3 × 10^5^ CD11b+ cells in culture.

### In Vivo GM-CSF Neutralization and Flow Cytometry

Mice were randomly assigned to receive anti–GM-CSF neutralizing antibody (1 mg/mL in sterile PBS, 505407 BioLegend, *n* = 5 mice [10 eyes]) or rat IgG (1 mg/mL in sterile PBS, 6-001-A; R&D Systems, Inc., *n* = 5 mice [10 eyes]) three times per day at 8 AM, 1 PM, and 6 PM for 7 days. Each treatment consisted of 2 μL of anti–GM-CSF or rat IgG administered topically via micropipette. Mice were housed in the CEC for a total of 7 days. To compare treatment versus control, corneal fluorescence staining (CFS) was blindly scored on days 0, 3, and 7 according to the NEI CFS scoring rubric (based on a scale of 0–15 points). On day 7, mice were euthanized and the cornea and conjunctiva were collected by first lifting conjunctiva from the fornix with jeweler's forceps and making incisions along both bulbar and palpebral insertion points with Vannas scissors. Then, a 30-gauge needle was used to enter the anterior chamber anterior to the limbus, and the cornea was excised. Draining lymph nodes were collected for flow cytometry analysis as described above, with the following changes. Lymph node cells were stained with PE/Cy5-conjugated anti-CD4 (eBioscience), fixed and permeabilized using a commercially available fixation/permeabilization kit (eBioscience) and then stained with FITC-conjugated anti–IL-17A (eBioscience). Cornea and conjunctival samples were digested with deoxyribonuclease and collagenase and stained with FITC conjugated anti-CD11b (eBioscience) and PE/Cy5 conjugated MHCII (BioLegend). Each flow cytometry sample consisted of tissue from one animal (*n* = 5 mice per treatment group).

### Statistical Analysis

Data normality was evaluated using graphing software (Prism 5.0; GraphPad Software, Inc., La Jolla, CA, USA). The Student's *t*-test or Mann-Whitney *U* test was used to determine statistical significance, with *P* < 0.05 considered statistically significant. Results are presented as the mean ± standard error of the mean.

## Results

### Expression of GM-CSF at the Ocular Surface Is Upregulated in DED

**Figure 1:**
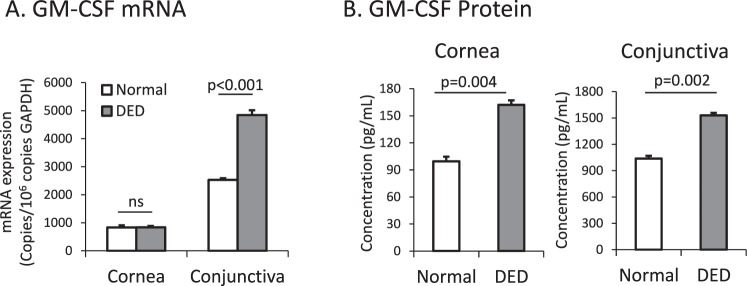
Expression of GM-CSF is upregulated in the cornea and conjunctiva of DED mice. (**A**) Granulocyte-macrophage CSF mRNA and (**B**) protein was assessed in the cornea and conjunctiva of normal and DED mice using real-time PCR and ELISA, respectively (*n* = 6 mice per group; data from one experiment of two is shown).

To investigate the role of GM-CSF in DED, we first evaluated the expression of GM-CSF at the ocular surface in normal and DED mice. Using real-time PCR, we found that mRNA levels coding for GM-CSF are significantly upregulated in the conjunctiva (normal: 2532±77 copies/10^6^ copies GAPDH versus DED: 4843 ± 170 copies/10^6^ copies GAPDH, *P* < 0.001, Fig. 1A,) but not the cornea (normal: 834 ± 56 copies/10^6^ copies GAPDH versus DED: 837±32 copies/10^6^ copies GAPDH, *P* > 0.05, Fig. 1A) of DED mice compared to normal mice. Using an ELISA assay, we found that the level of GM-CSF protein is significantly upregulated in the cornea (normal: 99±14 pg/mL versus DED: 162±19 pg/mL, *P* = 0.004, Fig. 1B) and conjunctiva (normal: 1038 ± 30 pg/mL versus DED 1530 ± 26 pg/mL, *P* = 0.002, Fig. 1B) of DED mice as compared to normal controls.

### Frequencies of GM-CSF–Producing Th17 Cells Increase in DED

**Figure 2:**
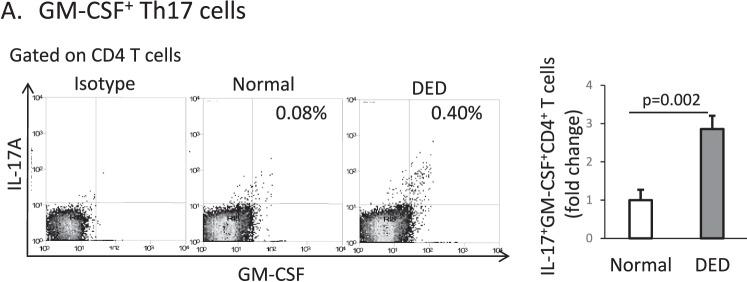
The frequency of GM-CSF+ Th17 cells is increased in DED mice. Flow cytometry analysis showing GM-CSF+IL-17+CD4+ T cells in the lymph nodes of normal (*white bar*) and DED (*gray bar*) mice (gated on CD4+; *n* = 6 mice per group; data from one experiment of two is shown).

Given that Th17 cells have been identified as one of the predominant effector immune cells in DED, we next sought to determine whether Th17 cells are a source of GM-CSF in DED. Using flow cytometry, we found that the frequency of GM-CSF+ Th17 cells in the draining lymph nodes of DED mice is significantly increased compared to normal mice (2.9 ± 0.12-fold increase in DED versus normal, *P* < 0.002, Fig. 2).

### Granulocyte-Macrophage CSF Blockade Suppresses CD11b+ Cell Maturation, Proliferation, and Migration In Vitro

**Figure 3:**
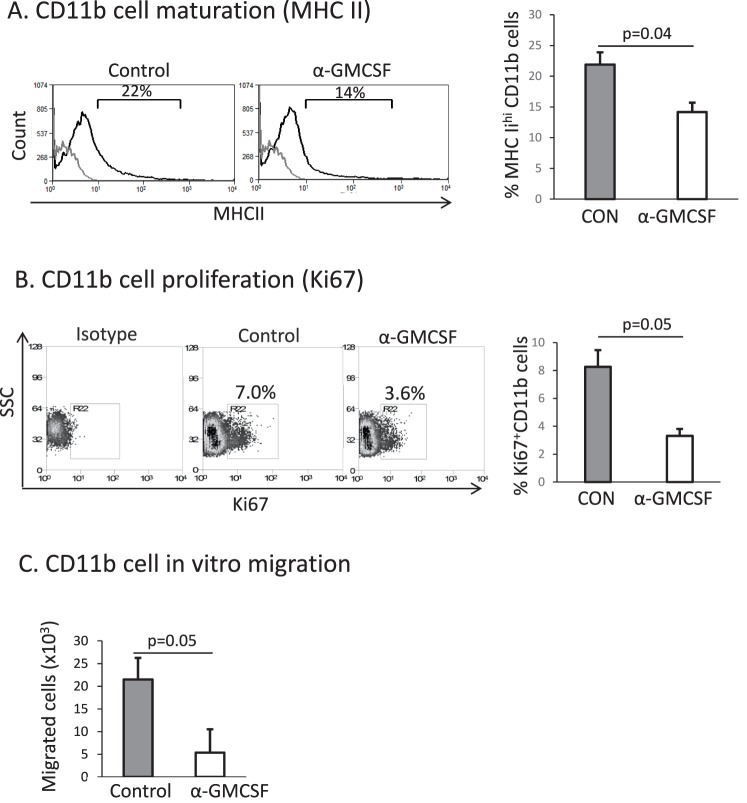
Blocking T cell–derived GM-CSF reduces CD11b+ cell maturation, proliferation, and migration in vitro. We isolated CD4+ T cells from the draining lymph nodes of DED mice and stimulated the cells with anti-CD3 antibody overnight. Culture supernatant was then incubated with either anti–GM-CSF or IgG control antibody and then added to a CD11b+ cell culture derived from naïve mice. Flow cytometry analysis shows MHC-II expressing (**A**) CD11b+ cells and (**B**) Ki67+ cells 24 hours after coculture (*gray line*: isotype control). In a transwell migration assay, GM-CSF neutralized or control supernatant was cultured with naïve CD11b+ cells (*upper well*). One hour later, CD11b+ cells that migrated to the lower well were enumerated. (Each group run in triplicate, data from one experiment of two is shown). CON, IgG isotype control; α-GM-CSF, anti–GM-CSF.

We next investigated the effect of T cell–derived GM-CSF on CD11b+ cells in vitro. CD4+ T cells isolated from DED mice were stimulated with PMA and ionomycin overnight, after which the culture supernatant was treated with either GM-CSF neutralizing antibody or IgG control, and then introduced to a cell culture consisting of CD11b+ cells derived from naïve mice for 24 hours. Using flow cytometry, we found that GM-CSF neutralization led to a significant decrease in the number of MHC-II^hi^ CD11b+ cells as compared to IgG control (IgG: 21.9 ± 2, anti–GM-CSF: 14.2 ± 1.6, *P* = 0.04, Fig. 3A). Neutralization of GM-CSF also led to a significant decrease in CD11b+ cell proliferation, as indicated by the number of CD11b+ cells that were positive for the cellular proliferation marker Ki-67 (IgG control: 8.3 ± 1.2, anti-GM-CSF: 3.3 ± 0.5, *P* = 0.05, Fig. 3B). Finally, using a transwell assay consisting of naïve CD11b+ cells (upper well) and anti–GM-CSF or IgG-treated CD4+ supernatant (lower well), we found that neutralization of GM-CSF inhibited the migration of CD11b+ cells in vitro (IgG: 21.5 ± 7.3 × 10^3^ cells, anti–GM-CSF: 5.4 ± 2.8 × 10^3^ cells, *P* = 0.05, Fig. 3C).

### Topical Blockade of GM-CSF Reduces CD11b+ Cell Maturation and Migration to the Ocular Surface

**Figure 4:**
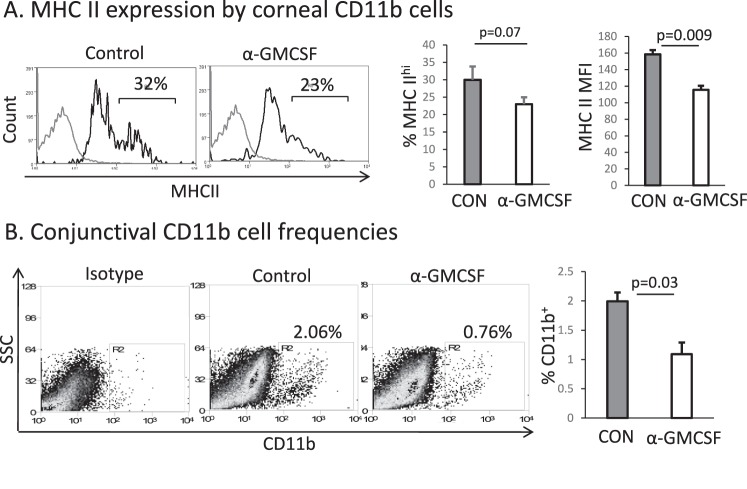
Topical neutralization of GM-CSF in a murine model of DED decreases CD11b+ cell maturation and migration to the ocular surface. We induced DED for 7 days and mice were treated topically 3 times daily with anti–GM-CSF or isotype control. Flow cytometry was used to determine frequencies and expression of MHC-II by (**A**) corneal CD11b+ cells and (**B**) frequencies of conjunctival CD11b+ cells. *Gray line*: isotype. *n* = 5 mice per group; data from one of two experiments is shown. CON, IgG isotype control; α-GM-CSF, anti–GM-CSF.

After demonstrating the effect of T cell–derived GM-CSF on CD11b+ cells in vitro, we next investigated the effect of topical administration of anti–GM-CSF neutralizing antibody on CD11b+ and Th17 cells in an in vivo model of DED. Compared to treatment with IgG control, neutralizing GM-CSF led to a decrease in the frequency of MHC-II^hi^ expressing corneal CD11b+ cells (IgG control: 30 ± 2, anti–GM-CSF: 23±1, *P* = 0.07) and a decrease in MHCII mean fluorescence intensity on corneal CD11b+ cells (IgG control:158 ± 3.5, anti–GM-CSF: 115 ± 2.5, *P* = 0.009, Fig. 4A). Neutralization of GM-CSF also led to a significant decrease in the number of conjunctival CD11b+ cells (IgG control: 2.00 ± 0.07, anti–GM-CSF: 1.09 ± 0.17, *P* = 0.03, Fig. 4B).

### Topical Treatment with anti–GM-CSF Improves Clinical Signs of DED

**Figure 5:**
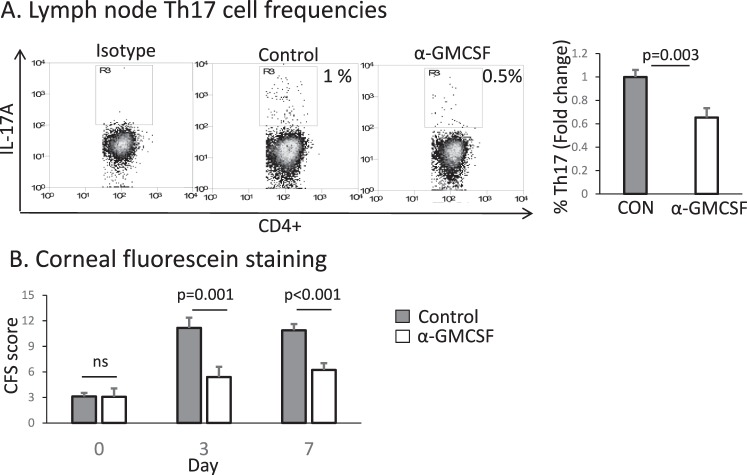
Topical treatment with anti–GM-CSF reduces pathogenic Th17 cells and improves clinical signs of DED. We induced DED for 7 days and mice were treated topically 3 times daily with anti–GM-CSF or isotype control. (**A**) Flow cytometry was used to determine frequencies of IL-17+ and CD4+ Th17 cells in the draining lymph nodes of treated versus control mice. (**B**) Clinical severity of DED after treatment with IgG isotype control (*gray*) versus anti–GM-CSF (*white*) was evaluated using CFS and the NEI scoring rubric (based on a scale of 0–15). Anti–GM-CSF significantly decreases corneal epitheliopathy compared to IgG control (*n* = 10 eyes per group; data from one of two experiments is shown). CON, IgG isotype control; α-GM-CSF, anti–GM-CSF.

We additionally evaluated whether topical blockade of GM-CSF had any effect on the frequencies of pathogenic Th17 cells that promote inflammation and disease severity. In the draining lymph nodes, we found significantly reduced frequencies of Th17 cells in mice treated with anti–GM-CSF compared with control-treated animals (0.6 ± 0.07-fold change in the anti–GM-CSF group compared to IgG control, *P* = 0.003, Fig. 5A).

Using the NEI standardized (0–15) CFS grading criteria, we also evaluated clinical disease after topical anti–GM-CSF treatment. There was no difference between the treatment and control groups on day 0 (IgG control: 3.1 ± 0.5, anti–GM-CSF: 3.1 ± 1.0, *P* > 0.05); however, mice treated with anti–GM-CSF had significantly lower CFS scores compared to the control group on both days 3 (IgG: 11.2 ± 1.2, anti–GM-CSF: 5.4 ± 1.2, *P* = 0.001) and 7 (IgG: 10.9 ± 0.7, anti–GM-CSF: 6.2 ± 0.9, *P* < 0.001, Fig. 5B).

## Discussion

T helper 17 cells have been shown to play an important role in multiple immune-mediated inflammatory conditions including rheumatoid arthritis, experimental autoimmune encephalomyelitis, inflammatory bowel disease, and dry eye disease.^8,19^ T helper 17 cells are characterized by their ability to produce several cytokines including GM-CSF, which has drawn attention as a key component of disease pathogenesis in a number of Th17 cell–associated conditions. Granulocyte-macrophage CSF acts on both circulating monocytes and tissue macrophages,^20^ and recent evidence suggests that GM-CSF promotes disease by stimulating these monocytic cells to produce inflammatory cytokines such as IL-1β, IL-6, and IL-23, the latter two being required for Th17 cell induction and pathogenicity.^14,21,22^ In the present study we show an additional mechanism by which Th17 cell–derived GM-CSF acts on CD11b+ monocyte/macrophages to promote disease, and that neutralization of GM-CSF can reduce these effects, yielding clinical improvement in a murine model of DED.

To investigate the role of GM-CSF in DED, we first evaluated the expression of GM-CSF in the cornea and conjunctiva of normal and DED mice. There was no difference in mRNA levels of GM-CSF between normal and DED corneas, but there was a significant upregulation of GM-CSF mRNA in the conjunctiva of DED mice (Fig. 1A). T helper 17 cells are known to readily infiltrate the conjunctiva in DED, but very few enter the peripheral cornea,^23,24^ suggesting that one possible explanation for the observed difference in mRNA expression between the cornea and conjunctiva is the presence of GM-CSF–producing Th17 cells in the conjunctiva of DED mice. We also found that protein levels of GM-CSF were significantly upregulated in the conjunctiva of DED mice, which is consistent with Th17 cells acting as a source of GM-CSF. There was also a significant increase in GM-CSF protein in the corneas of DED mice (Fig. 1B), which similarly may be derived from Th17 cells that have infiltrated the adjoining conjunctiva. To further evaluate Th17 cells as a source of GM-CSF in DED, we identified and enumerated GM-CSF–producing Th17 cells in our disease model. We found that the frequency of GM-CSF+ Th17 cells significantly increases in DED, which is consistent with previous reports suggesting that Th17 cells may act as a primary source of GM-CSF at the ocular surface in DED.^9,25^

Granulocyte-macrophage CSF promotes inflammation and immunity through multiple mechanisms.^26^ These include its classical role in promoting myeloid cell proliferation and development, as well as through promoting mature macrophage cytokine production^27^ and antigen-presenting cell function^28^ and chemotaxis.^29,30^ Given that CD11b+ cells function as antigen-presenting cells and are upregulated in DED,^9,23^ we evaluated the effect of CD4+ T cell–derived GM-CSF on CD11b+ cells and found that anti–GM-CSF treatment significantly decreased CD11b+ cell maturation and proliferation compared to IgG control–treated supernatant (Fig. 3). Using an in vitro transwell assay, we also found that neutralization of GM-CSF suppressed CD11b+ migration. This is consistent with past work demonstrating that GM-CSF is directly chemotactic for macrophages.^29,30^ In vivo, GM-CSF may also indirectly modulate immune cell migration by stimulating monocytes to produce proinflammatory cytokines (such as TNF-α) which then upregulate chemokines and integrins that facilitate cell migration.^31^

Given these in vitro findings, we next evaluated the effect of topical GM-CSF neutralization in DED mice. We found that topical blockade of GM-CSF significantly decreases DED severity as measured by CFS scoring (Fig. 5). Tissue analysis showed that GM-CSF blockade reduced CD11b+ cell migration to the ocular surface as well as the expression of MHC-II by those cells. We also evaluated the induction of the Th17 immune response after topical treatment, and found that anti–GM-CSF significantly reduced the frequency of Th17 cells generated as compared to control. Since treatment was administered locally at the ocular surface and T cells themselves do not express the GM-CSF receptor,^32,33^ it is possible that this finding is a downstream result of the decreased number of CD11b+ cells recruited to the ocular surface and suppression of MHCII expression by those cells. It is also possible that topical administration of anti–GM-CSF also suppresses MHCII maturation on CD11b+ cells located beyond the ocular surface, such as in the draining lymph nodes, as topically administered neutralizing antibodies do have some systemic distribution.^34^ These changes in CD11b+ cell frequency and maturation then lead to subsequent suppression of Th17 cell induction and the observed improvement in corneal epitheliopathy following GM-CSF neutralization.

In conclusion, we demonstrate an increased frequency of GM-CSF–producing Th17 cells in DED which leads to upregulation of GM-CSF at the ocular surface. Blockade of GM-CSF reduces CD11b+ cell infiltration of the ocular surface and CD11b+ cell acquisition of MHCII, resulting in decreased ocular surface inflammation and clinical signs of disease. Taken together, our data suggest that one mechanism by which Th17 cells drive immune-mediated diseases is through their production of GM-CSF, which amplifies disease through the recruitment and activation of CD11b+ immune cells.
